# Zein Microparticles and Nanoparticles as Drug Delivery Systems

**DOI:** 10.3390/polym14112172

**Published:** 2022-05-27

**Authors:** Iolanda De Marco

**Affiliations:** Department of Industrial Engineering, University of Salerno, Via Giovanni Paolo II, 132, 84084 Fisciano, Salerno, Italy; idemarco@unisa.it

**Keywords:** zein, drug delivery systems, microparticles, nanoparticles, controlled release

## Abstract

Zein is a natural, biocompatible, and biodegradable polymer widely used in the pharmaceutical, biomedical, and packaging fields because of its low water vapor permeability, antibacterial activity, and hydrophobicity. It is a vegetal protein extracted from renewable resources (it is the major storage protein from corn). There has been growing attention to producing zein-based drug delivery systems in the recent years. Being a hydrophobic biopolymer, it is used in the controlled and targeted delivery of active principles. This review examines the present-day landscape of zein-based microparticles and nanoparticles, focusing on the different techniques used to obtain particles, the optimization of process parameters, advantages, disadvantages, and final applications.

## 1. Introduction

Zein is the major storage protein of corn first identified in the nineteenth century. It constitutes 44–79% of the endosperm protein depending on the variety of corn and the technique used for its extraction [1]. Being a natural material, it can be easily extracted from corn and is extensively available in nature [2]. Zein belongs to the category of prolamins and, from the biological point of view, it is a mixture of four components (α, β, γ, and δ) with different peptide chains, molecular size, and solubility [3]. Without any doubt, α-zein is the most abundant constituent of the mixture, accounting for about 70–80% of the whole zein, followed by γ-zein, which constitutes 10–20% [4,5]. It is the main component of commercially available zein, which is available in two forms, yellow and white zein. The former contains a high concentration of xanthophyll pigments and has a purity of about 90%. In contrast, white zein is obtained by decolorization of the yellow protein, has a negligible amount of xanthophylls, and has a purity higher than 96% [6]. Zein is a hydrophobic protein: it is poorly soluble in water alone and is soluble in aqueous ethanol, aqueous acetone, and some organic solvents [7].

The hydrophobicity, biodegradability, and biocompatibility of zein have been exploited in various fields, such as food coating [8] and packaging [9], adhesives [10], coatings [11], in the textile sector [12], in the biomedical sector and tissue engineering [13], and in the pharmaceutical industry [14]. Being a versatile polymer, zein is processed to obtain different shapes, such as particles [15], films [16], membranes [17], and scaffolds [18], which have been used for different applications. Zein films obtained by solvent casting, extrusion, or compression molding are generally used in the biomedical field, but because of the poor ability of zein to act as a water barrier, plasticizers or crosslinkers are necessary, or, in some cases, hybrid films are used incorporating other biopolymers [19,20]. Zein nanofibers produced by electrospinning are used to obtain scaffolds for tissue engineering [21] or food packaging applications [22].

Recently, zein has been utilized as a carrier for oral drug delivery systems [23] because it guarantees enhanced bioavailability, preparing sustained-release dosage forms, and targeting/protecting drugs [24]. Different reviews have been published on this topic, generally focused on the different morphologies obtained [13,24,25]; in some cases, the use of zein in combination with other substances has been deepened [7].

This review was focused on the attainment of zein in the form of micro and nanoparticles; unlike the previous reviews, the papers were classified according to the different processing techniques used to obtain the particles. The main limitation in the use of zein is that it is administered in the form of multiparticulate delivery systems associated with low drug loadings, high need of excipients, huge production costs, and use of advanced technologies [26].

## 2. Microparticles and Nanoparticles Production

Micronization processes are based on reducing the average diameter of a solid material. In the pharmaceutical sector, reducing dimensions down to the micro- or nanoscale is desirable since an increase in the bioavailability of the active ingredients is obtained in correspondence with a higher surface/volume ratio; in fact, active ingredients are often poorly soluble in water. The techniques used to obtain microparticles (MP) and nanoparticles (NP) can be classified into two main categories: top–down and bottom–up techniques [27,28]. In top–down processes, large particles are broken into smaller particles through milling, grinding, high-pressure homogenization, or other mechanochemical methods [29,30,31]. In bottom–up techniques, the active principle ingredient (API) is solubilized in an organic solvent and precipitates in the form of microparticles and nanoparticles by adding an antisolvent: the most commonly used techniques are the liquid antisolvent method, supercritical fluids processes, spray drying and emulsification, and solvent evaporation [32]. Bottom–up approaches are preferred in the case of thermolabile solutes, such as pharmaceuticals, because in top–down techniques, high energy input is involved, and a high amount of heat is generated, making the processing of thermolabile materials problematic. Almost all of the papers involving zein micronization consider bottom–up techniques.

In the last twenty years, research in the pharmaceutical sector has focused mainly on obtaining MPs and NPs of coprecipitates consisting of an API and a suitable polymeric carrier. In this way, it is possible to protect the active principle from light or oxidizing agents, mask unpleasant odors or tastes, and modulate the release of the active principle by suitably varying the type of carrier used and the polymer/drug ratio [33]. Zein alone or covered with other polymers was precipitated in some papers in the form of MPs and NPs [34,35,36,37,38,39]. The operating conditions were optimized to obtain the proper morphology, mean size, and particle size distribution. However, those papers were not analyzed in depth, considering that the present review paper was focused on the attainment of coprecipitated particles constituted by zein + API. This review is organized into subsections in which the published papers are classified as follows: (a) liquid antisolvent process; (b) supercritical antisolvent processes; (c) coacervation; (d) other techniques.

### 2.1. Liquid Antisolvent Process

The liquid antisolvent (LAS) process is the one mostly used to coprecipitate zein with an API in the form of microparticles and nanoparticles. In numerous cases, a co-carrier or a covering polymer has been used in combination with zein to confer the proper characteristics to the obtained material. The LAS process is based on the dissolution of zein and an API in a suitable organic solvent (generally aqueous ethanol), subsequently added to an aqueous phase containing a surfactant. The organic solvent is usually removed using rotary evaporation, stirring at high velocity, freeze-drying, or spray-drying. The main results obtained with the optimal operating conditions are schematized in Table 1.

**Figure 1:**
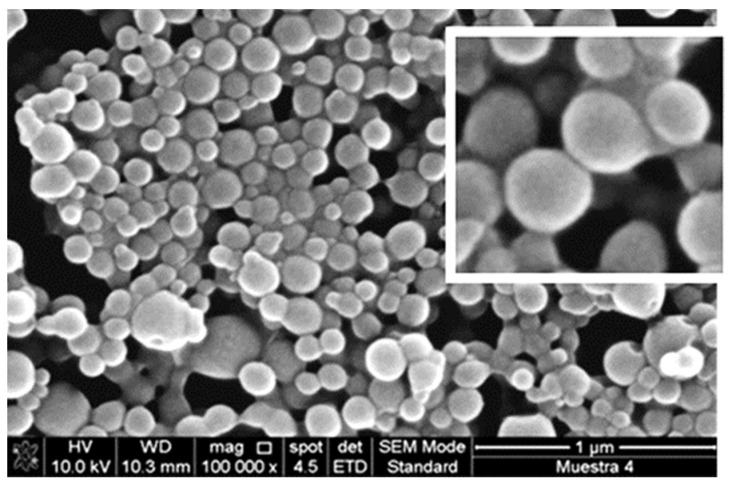
Nanoparticles of zein/resveratrol. Adapted with permission from [95]. Copyright 2015 American Chemical Society.

From the results reported in Table 1, it is clear that zein + API powders are obtained almost in all cases in the form of nanoparticles using the LAS method. In some cases, particle size distributions are broad, but in other cases, monodisperse NPs are obtained. An exemplificative SEM image of monodisperse nanoparticles obtained using liquid antisolvent precipitation is reported in Figure 1. The particles are constituted by resveratrol, a polyphenol with antioxidant, antiaging, and anticancer properties, loaded into zein. The nanoparticles obtained are constituted by a homogeneous population of spherical particles with a smooth surface [95].

**Figure 2:**
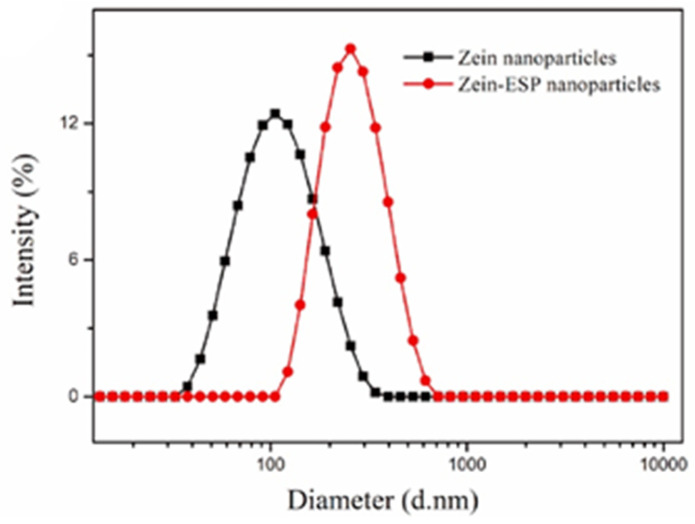
Particle size distribution of zein and zein ethanol-soluble polysaccharide NPs measured by DLS. Adapted with permission from [56]. Copyright (2022) Elsevier.

The dimensions of the particles are determined either with the aid of software based on image analysis or, more correctly, through dynamic light scattering (DLS). An exemplificative distribution obtained using a DLS particle size analyzer is reported in Figure 2.

The LAS technique has been used to process different materials with anti-inflammatory, chemotherapeutic, and antioxidant properties. In some cases, the in vitro cytotoxicity of the processed NPs against cancer cells was evaluated with encouraging results [44,46,62,68,70,85,93,99,102]. Moreover, in vivo studies on rats [74,92,95] or on human volunteers [97] were performed in some cases. When a comparative investigation of different carriers was attempted, zein showed a greater ability to retain the active compound effectively. Indeed, Gagliardi et al. [99] coprecipitated rutin, a polyphenolic bioflavonoid characterized by peculiar antioxidant properties, using poly(lactic-co-glycolic acid) (PLGA) or zein as the carrier. They observed that PLGA nanoparticles showed a poorer ability to retain rutin with respect to zein nanosystems.

**Figure 3:**
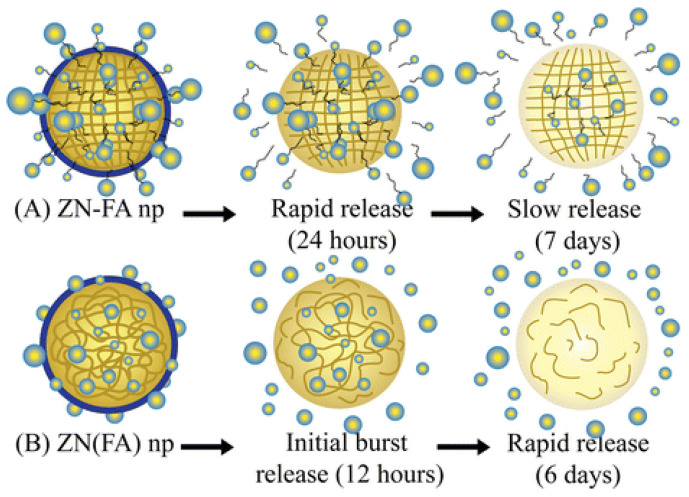
Release mechanisms of FA from NPs: covalently linked (**A**) and physically entrapped (**B**). Reprinted with permission from [69]. Copyright 2017 Springer.

Other authors compared the performance in terms of drug release of differently obtained systems based on the use of zein. Indeed, Chuacharoen and Sabliov [69] compared zein NPs covalently linked to folic acid (FA) (through the reaction between the carboxyl group of FA and the primary amino group of zein) and zein NPs with physically entrapped FA. In the former case, the particles were able to sustain the release of the active principle and target cancer cells overexpressing folate-binding receptors. In contrast, in the latter case, zein NPs controlled the release of the bioactive substance without targeting cancer cells. The proposed release mechanisms are sketched in Figure 3.

Contado et al. [98] coated resveratrol-loaded zein–pectin nanoparticles with Eudragit S100 to avoid the degradation of the dissolved drug in the gastrointestinal tract; they obtained nanoparticles with a mean diameter (MD) of 250 nm with targeted delivery in the colon tract. Indeed, it is well-known that Eudragit polymers are pH-sensitive polymers and, therefore, a specific Eudragit can be chosen depending on the type of desired drug release [103].

The main drawback of the LAS process is the presence of a high amount of ethanol; this can limit the use of the LAS technique because of the high costs related to the removal of the solvent itself and the residual solvent which can be contained in the precipitated powders.

### 2.2. Supercritical Antisolvent Process

An innovative process that has been used to coprecipitate a carrier with active principles is supercritical antisolvent precipitation (SAS) [104,105]. In this process, a liquid antisolvent is substituted with carbon dioxide in supercritical conditions. Therefore, this process is based on two prerequisites:

- the carrier (zein) and the drug have to be soluble in an organic solvent but insoluble in the mixture formed by this organic solvent and supercritical carbon dioxide (scCO_2_);
- the organic solvent and scCO_2_ have to be miscible under the process conditions.

Concerning the liquid antisolvent process, in the SAS process, the peculiarities of scCO_2_ can be exploited [106]. As a consequence, the size of the obtained particles can be easily controlled at the micro- and nanoscale by varying the process parameters, such as pressure, temperature, total concentration of the liquid solution, and ratio between the zein and the drug; moreover, complex post-process operations to separate the solvent and the antisolvent are not necessary, considering that in correspondence of the ambient conditions of temperature and pressure carbon dioxide is in the gaseous state whereas the organic solvent is a liquid. It was previously demonstrated that the use of different carriers in the SAS process leads to different drug releases: hydrophilic polymers such as polyvinylpyrrolidone can be used to obtain an increase in the drug dissolution rate, whereas hydrophobic carriers, such as zein, are the right choice to obtain a prolonged release of the active principle [107].

**Figure 4:**
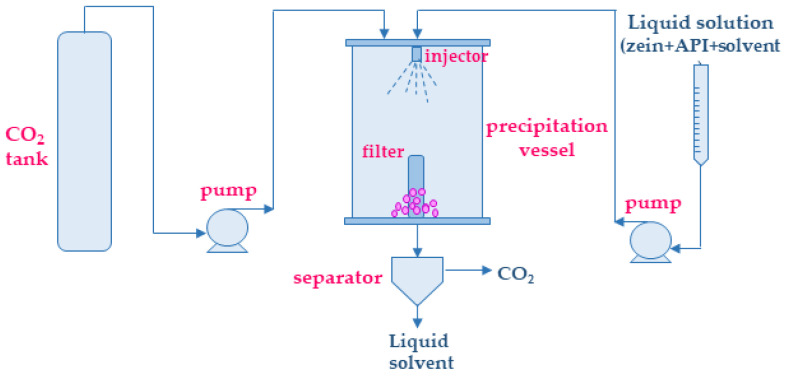
Sketch of a typical supercritical antisolvent precipitation plant.

In Figure 4, a typical SAS plant is sketched. It comprises a carbon dioxide tank, a liquid solution burette, two pumps (one for CO_2_ and one for the liquid solution), a precipitation vessel, and a separator. The precipitation vessel is the heart of the plant and is equipped with an injector mounted in the top part of the chamber and a filter at the bottom from which the particles can be recovered after depressurization.

In Table 2, a list of the active ingredients coprecipitated with zein using the SAS process and the operating conditions chosen by the different research groups, and the main results obtained are reported.

It can be noted that using the SAS process, zein, in general, precipitates in the form of microparticles rather than nanoparticles and that the organic solvents most frequently used are DMSO or aqueous ethanol. The pressure ranges from 8.0 to 16.0 MPa, the temperature—from 30 to 55 °C. The zein/API weight ratio can vary in a wide range, but typically ratios in the range of 5:1–20:1 are preferred by different authors. The selected active principles belong to different categories, such as vitamins [109,113], antibiotics [108], anti-inflammatory drugs [111], anticancer drugs [112], or antihistamine drugs [110]. When the dissolution rate of the active principle coprecipitated with zein was compared with the dissolution rate of the pure API, a clear effect of prolonging the release was observed. For example, in the case of amoxicillin [108], complete API dissolution was achieved in almost 3 days and, therefore, the formulation can be used for “long-term antibiotic therapy”. In the case of 10-hydroxycamptothecin (HCPT) nanocrystals coprecipitated with zein microparticles [112], there was an initial burst corresponding to the fast release of the 50% of the drug in the first 10 h (attributed to the immediate dissolution and release of the HCPT located near the surface of the particles), followed by slow dissolution of the API corresponding to the release of the 70% of the drug in 82 h (because of the drug entrapped into the zein microspheres). A bimodal release was also observed in the case of antihistamines [110]: an immediate release of a small amount of the drug (22%) useful to rapidly relieve the symptoms associated with allergy, followed by a prolonged release of the remaining drug (that was completely dissolved in 36 h), which reduces the number of administrations throughout the day.

The SAS process has also been used to combine zein with other substances to deliver active principles. For example, Liu et al. [116] prepared nanospheres constituted by zein decorated with folic acid to obtain targeted delivery of HCPT. Indeed, HCPT is a promising natural anticancer ingredient characterized by poor aqueous solubility and in vitro and in vivo instability. The authors added folic acid to obtain a sustainable and targeted delivery system, enhancing the intracellular uptake of HCPT within cancerous cells. Compared to zein nanoparticles, folic acid/zein nanoparticles were smaller (in the range of 350–820 nm) and had a higher stability; moreover, folic acid/zein conjugates have the potential to selectively target tumor cells, with an associated reduction in nonspecific toxicity in the normal cells.

Palazzo et al. [117] used a supercritical-based process named supercritical assisted injection in a liquid antisolvent (SAILA) to entrap luteolin in zein microparticles. The process is based on the continuous injection of an expanded liquid constituted by an organic solvent, zein, API, and scCO_2_ in an aqueous solution, which has the role of the antisolvent. Therefore, unlike the SAS process in which scCO_2_ is the antisolvent, in the SAILA process, scCO_2_ is a co-solute. The authors identified the best operating conditions at a pressure of 10.0 MPa, a temperature of 40 °C, and a zein/luteolin ratio of 20:1. In correspondence with these conditions, they obtained microparticles with an MD of 1.20 μm and an entrapment efficiency of 82%.

The main advantage of the techniques based on the use of scCO_2_ lies in controlling the size of the particles as the operating conditions vary. In general, the particle size increases with increasing concentration of the liquid solution and decreasing pressure. Another significant advantage lies in the absence or presence of traces below the permitted limits of the solvent residue in the powders. On the other hand, the investment costs for the construction of plants and the operating costs of the processes themselves are higher than those of the LAS process due to high operating pressures.

### 2.3. Coacervation

The coacervation process involves no harsh solvents or high temperatures. The process consists of the separation of solutions into colloidal systems with two liquid phases: one, called coacervate, is rich in polymer and another phase is without the polymer. Coacervation can be simple when it involves the use of a single polymer or complex when two natural biopolymers of opposite charges are involved.

Simple coacervation has been used to prepare zein microspheres conjugated with different drugs, such as heparin [118], gitoxin [119], chemotherapeutic agents [120], and antigens for the preparation of vaccines [121,122] or DNA [123]. For example, Wang et al. [118] prepared a drug-eluting coating film containing zein + heparin microspheres with slow API release; indeed, about 55% of the entrapped heparin was released after 20 days. The film constituted by microparticles showed adequate anticoagulation and improved hemocompatibility. Susuki et al. [120] conjugated zein microspheres with some antitumor drugs, such as mitomycin C, daunomycin hydrochloride, and peplomycin sulfate, obtaining sustained API release systems to be used in selective cancer chemotherapy by oral or intratumoral administration. Regier et al. [123] prepared zein/DNA nanoparticles with an MD ranging from 158 to 397 nm depending on the zein/DNA ratio. The authors demonstrated a sustained plasmid release for at least 7 days, with a minimal initial burst. Zein/DNA nanospheres showed robust biocompatibility. They can be fine-tuned for specific applications including oral gene delivery, intramuscular delivery, and in the fabrication of tissue engineering scaffolds.

Complex coacervation has been used, for example, to prepare zein and chitosan coacervates, considering the zein/chitosan ratio, solid/liquid ratio, and pH. Indeed, Li et al. [123] studied the morphology and encapsulation efficiency of curcumin in the correspondence of different coacervation conditions. The in vitro release study showed that the stronger the zein–chitosan interaction was, the less the amount of curcumin released from the nanoparticles was. The API at the optimized operating conditions has a slight burst effect followed by a slow release.

The main advantages of coacervation are the rapidity of the process and the absence of solvents. In contrast, the drawbacks are the potential toxicity of the crosslinkers and the difficulty of controlling the coagulation step.

### 2.4. Other Techniques

**Figure 5:**
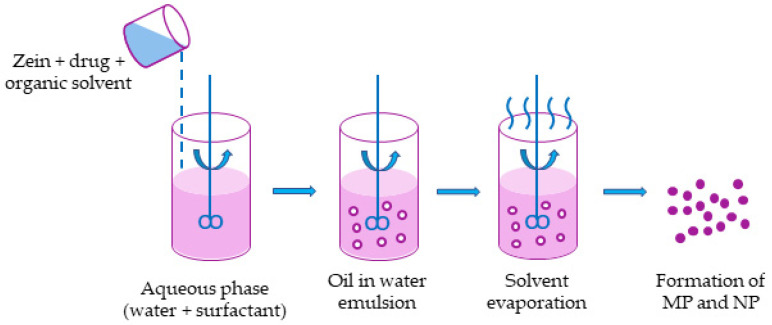
Schematic representation of emulsification followed by solvent evaporation.

Zein has also been processed using other techniques. In the emulsification and solvent evaporation method, an oily phase constituted by zein + API + organic solvent is emulsified into an aqueous phase in which a surfactant is dissolved. The system is continuously stirred to reduce the droplet size that forms the emulsion. Then, the organic solvent is evaporated under vacuum, and microparticles and nanoparticles precipitate in the second step. In Figure 5, a schematic representation of the process is reported.

For example, aceclofenac sodium, a nonsteroidal anti-inflammatory drug used extensively in treating rheumatoid arthritis and osteoarthritis, was processed with this technique [124]. The disperse phase was prepared by dissolving the drug and zein in 90% alcohol. This solution was added to the continuous phase (sesame oil) containing 0.5% Span 80 as an emulsifying agent. MPs with an MD in the range of 136–174 μm were obtained; the EE varied from 11.6 to 26.1% depending on the zein/drug ratio. In vitro release studies were attempted simulating gastric and intestinal fluids: a sustained release up to 72 h was detected. Biocompatibility of the zein microspheres was evaluated through in vitro cytotoxicity studies using fibroblast cells from the explant tissue. Karthikeyan et al. [125] used the same method to prepare zein microspheres charged with aceclofenac, metformin, and promethazine. The three active principles were chosen as examples of hydrophobic, hydrophilic, and amphiphilic drugs. The average particle size of the different zein/API microspheres was found to be 68–136 μm depending on the drug. The EE was around 20–25% depending on the zein/API ratio. The higher the ratio, the higher the EE.

A novel approach proposed by some authors is the preparation of zein microstructures by electrospinning and spray-drying (SD). For example, Coelho et al. [126] incorporated vitamin B12, the most chemically complex and the largest molecule among all the vitamins, into zein. The microparticles obtained by electrospinning had a size of around 3 μm and an EE of 91%. In contrast, wrinkled coprecipitated microparticles were obtained by SD with an average size of 6.4 μm and an EE of 95%. In vitro release tests revealed that a controlled release profile characterized the API and that vitamin B12-loaded zein microstructures produced by electrospinning have a slower release profile than the structures obtained by spray-drying. Mahalakshmi et al. [127] encapsulated β-carotene in zein at the microlevel using SD (with an MD in the range of 1.4–2.5 μm) and at the nanolevel using electrospraying (with an MD of 600–900 nm). Electrospraying proved to have higher encapsulation efficacy than SD. In vitro simulated gastrointestinal stability studies showed that the release of encapsulated β-carotene from NPs is faster than from MPs due to the larger surface area interacting with the release medium. SD was also used by Sousa et al. [128] to develop microspheres of PLGA and zein for amoxicillin and indomethacin delivery. In the case of amoxicillin, they obtained MPs with a mean diameter in the range of 9.4–38.3 μm depending on the PLGA/zein ratio with an EE up to 51%; in the case of indomethacin, the mean diameter varied in the range of 5.6–38.5 μm, and the EE was higher (up to 99%). In vitro release studies revealed a sustained-release pattern for all the formulations. De Sousa et al. [129] obtained tetracycline-loaded microparticles made of PLGA and zein using SD for teeth preservation therapy (chronic periodontitis). They obtained a product with a different EE depending on the PLGA/zein ratio. In vitro drug release studies showed a sustained release of tetracycline over 700 h in water (30 days), which is a period that would guarantee the treatment of a long course of periodontitis. The antimicrobial activity against *Staphylococcus aureus* was also evaluated.

Nanoprecipitation was successfully used by de Souza Tavares et al. [130] who nanoencapsulated ellagic acid, a compound with antioxidant and antimicrobial activities, into zein nanoparticles. They obtained spherical, non-aggregated, smooth-surface particles under 370 nm in diameter with relevant inhibitory and bactericide activity against *S. aureus* and *P. aeruginosa*. The obtained system can represent a suitable alternative to prevent and treat infectious attributed to Gram-positive and Gram-negative bacteria; moreover, it was demonstrated that the antioxidant effect was preserved for 24 h, such as required in skin repairing and topical treatments. Weissmueller et al. [131] used a flash nanoprecipitation process to encapsulate some APIs, such as vitamin E acetate and anticholera quorum-sensing modulator CAI-1 ((S)-3-hydroxytridecan-4-one). They obtained particles with a diameter less than 100 nm with high loading for both active principles. The stability of the obtained particles in the simulated intestinal fluid was demonstrated for 24 h. Zein nanoparticles incorporated with digoxin, a drug used to treat heart failure, were obtained by nanoprecipitation and then charged in an alginate film to prepare a buccal drug delivery system [132]. Digoxin was successfully encapsulated into zein nanoparticles with an EE of 91% and a mean size of 87 nm. It was also demonstrated that the mucoadhesive film incorporated with zein + API nanoparticles presented a controlled swelling profile and mechanical properties compatible with the application as a drug delivery system through the buccal mucosa.

Another process to reduce the use of organic solvents is acidification of a strong alkaline solution containing zein and an API, as proposed by Yuan et al. [133], who prepared pH-driven zein/tea saponin composite NPs containing curcumin. The process is based on the principle that the solubility of dissolved zein + API decreases during the acidification process and forms a sphere spontaneously to avoid the polar environment. The obtained spherical NP had an EE equal to 84% and high bioaccessibility with respect to free curcumin. Sabra et al. [134] synthesized amphiphilic protein copolymers via a carbodiimide coupling reaction for the tumor-targeted delivery of rapamycin and wogonin, two anticancer drugs. The nanoplatform was composed of a hydrophobic zein core to encapsulate drugs with high EE, a hydrophilic lactoferrin corona to enhance tumor targeting and prolong systemic circulation of nanocarriers, and glutaraldehyde crosslinking to reduce the particle size and improve micellar stability. The particle size of the micelles was around 260–290 nm and the EE was higher in the case of wogonin than for rapamycin. In vitro release profiles revealed that wogonin release from micelles was biphasic, characterized by initial fast release of about 64% of the drug during the first 6 h followed by the second phase of very slow release with about 67.59% of WOG released after 24 h. On the contrary, rapamycin showed a very slow release (<20% drug release after 72 h) without a considerable initial burst effect. Moreover, this combined nanodelivery system maximized synergistic cytotoxicity of the two drugs in terms of tumor inhibition in MCF-7 breast cancer cells.

**Figure 6:**
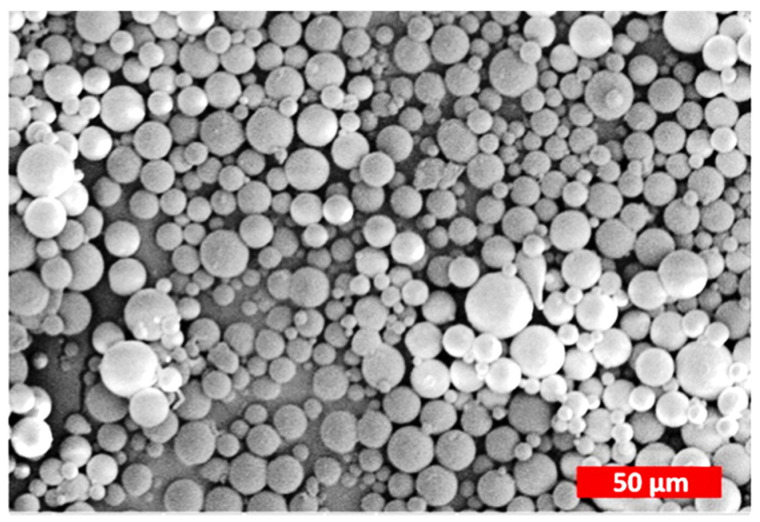
FESEM image of chitosan/zein nano-in-microparticles prepared by ionic gelation. Reprinted with permission from [137]. Copyright (2017) Elsevier.

In some cases, zein was not used as the carrier coprecipitated with the active principle but as the coating material. For example, Vozza et al. [135,136] used ionotropic gelation to encapsulate selenoamino acids (selenomethionine, methylselenocysteine, and selenocysteine) with antioxidant and anticancer properties into chitosan nanoparticles; the NPs were, then, coated with zein. At the best operating conditions in terms of the chitosan/zein ratio, they obtained particles with an MD of 271 nm and an encapsulation efficiency (EE) of 81% in the case of methylselenocysteine, particles with an MD of 377 nm in the case of selenomethionine and an EE of 80%, whereas in the case of selenocysteine, the MD was 262 nm and the EE was 79%. The analyses of the particles showed no cytotoxicity in Caco-2 cell lines and a sustained release of the APIs. Farris et al. [137] prepared chitosan/zein nano-in-microparticles constituted by a core of chitosan/DNA nanoparticles prepared by ionic gelation and further encapsulated in zein microparticles obtained using a water-in-oil emulsion. Well-defined micrometric particles were obtained, as reported in the FESEM image in Figure 6. Analyses such as DNA release profiles, site-specific degradation of the outer zein matrix, and in vivo transfection demonstrate that the formulated particles can improve oral gene delivery through enhanced protection and controlled release of the DNA cargo.

## 3. Conclusions

This review focused on the use of zein particles in the pharmaceutical field. Zein is a versatile polymer, which can be used alone, in blends with other polymers, and in shell–core systems constituting both the shell or the core polymer to obtain sustained-release drug delivery systems. Depending on the process and the operating conditions, zein is precipitated in the form of microparticles or nanoparticles. The liquid antisolvent process generally generates powders in the nanometric range, whereas supercritical antisolvent precipitation gives microparticles. The traditional and largely employed LAS method has the advantage of simplicity and low costs. Conversely, the SAS method is characterized by lower amounts of organic solvents and high costs; for this reason, it has been prevalently applied only at a laboratory scale. Other processes, such as coacervation, emulsification, and solvent evaporation, or electrospinning, have also been used to obtain sustained-release powders.

## Figures and Tables

**Table 1:** Zein-based particles obtained using the liquid antisolvent process. AS/S, antisolvent/solvent; ATRA, all-trans-retinoic acid; β-CD, beta-cyclodextrin; Bor, bortezomib; BSA, bovine serum albumin; c, concentration in the liquid solution; C-28, human chondrocyte cells; CA, caffeic acid; Caco-2, human colon carcinoma cells; CS, chondroitin sulfate; CSA, carboxymethylated short-chain amylose; DOX, doxorubicin; DPPH, 2,2-diphenyl-1-picrylhydrazyl; DU145, prostate cancer cells; EC, ethyl cellulose; EE, encapsulation efficiency; EGCG, epigallocatechin gallate; ESP, ethanol-soluble polysaccharide; EtOH, ethanol; FA, folic acid; FD, freeze-drying; GA, gum arabic; GI, gastrointestinal; GLI, glibenclamide; Glim, glimepiride; GNA, gambogenic acid; GT, poly(anhydride)-thiamine conjugate; HA, hydroxyapatite; HAc, hyaluronic acid; HACAT, immortalized human keratinocyte cells; HeLa, highly stabilized immortalized tumor cells; HepG2, human liver cancer cells; HP-β-CD, 2-hydroxypropyl-beta-cyclodextrin; HT29, human colorectal adenocarcinoma cells; HT29-MTX, human colon cancer cells; Hya, hyaluronan; Indo, indomethacin; K562, chronic myelogenous leukemia cells; mPEG, methoxy poly(ethylene glycol); MCF-7, human breast cancer cells; MD, mean diameter; MP, microparticles; MW, molecular weight; NCTC2544, human keratinocyte cells; Nic, niclosamide; NCI/ADR-RES, ovarian tumor cells; NP, nanoparticles; PC3, prostate cancer cells; PDA, polydopamine; PEG, poly(ethylene glycol) 2000; PGA, propylene glycol alginate; PL, polydopamine–lecithin; PNS, *Panax notoginseng saponins*; PSA, polysialic acid; PTS, pterostilbene; Que, quercetagetin; Res, resveratrol; SC, sodium caseinate; SD, spray-drying; SGF, simulated gastric fluid; SIF, simulated intestinal fluid; SR, sustained release; Suc, succinic anhydride; SW480, colon cancer cells; T, temperature; Toc, tocopherol; TS, tea saponin; Vor, vorinostat; XG, xanthan gum; 4T1, mammary carcinoma cells; A549, human lung carcinoma cells.

Active Principle	Co-Carrier	Organic Solvent (*v*/*v*)	Zein/API (*w*/*w*)	Solvent Removal	Results	Reference
5-fluorouracil	–	Aqueous EtOH 75%	From 2:1 to 16:1	Stirring + FD	NPs with MD = 115 nm; EE up to 61%; burst release in the first 15 min + slow release for 24 h	[40]
Artemether	SC	Aqueous EtOH 50–90%	From 2.5:1 to 20:1	Stirring or rotavapor for 1–5 h	NPs with MD = 142–201 nm depending on the c and zein/API ratio; EE up to 62%; burst release in the first 30 min + SR for 1.5 h; intravenous administration in rats revealed an extension of the API’s mean residence time	[41]
Atorvastatin	–	Aqueous EtOH 80%	From 1:1 to 10:1	Stirring at 2000 rpm for 3 h + FD for 72 h	NPs with MD = 223 nm at a low zein/API ratio; MPs with MD = 1.1 μm at a high zein/API ratio; EE up to 67%; dissolution profile with a burst in the first 4 h + a SR	[42]
ATRA	–	Aqueous EtOH 66%	20:1	Stirring at 600 rpm for 12 h	NPs with MD = 103–401 nm depending on the c and kind of surfactant; EE up to 14%	[43]
ATRA	Phospholipids	Aqueous EtOH 70%	From 5:1 to 20:1	Homogenization at 24,000 rpm for 2 min + stirring at 600 rpm for 12 h	NPs with MD = 80–200 nm depending on the zein/API ratio; EE up to 60%; evaluation of in vitro cytotoxicity in A549 and CaCo2 cells	[44]
CA + FA	Chitosan	Aqueous EtOH 70%		Stirring + SD (inlet temperature of 100 °C)	NPs with MD = 172–254 nm depending on the CA amount, FA amount, chitosan/zein ratio; EE up to 64% for CA and 84% for FA; chitosan forms complexes with FA, whereas CA is encapsulated in the hollow core	[45]
Carvacrol	Lecithin	Aqueous EtOH 80%		Stirring at 500 rpm overnight	NPs with MD = 221–312 nm depending on the zein/API ratio; EE = 78%; 9% of the API released in 2 h (SGF conditions), up to 78% of the API (SIF conditions)—in 24 h; cytotoxicity against SW480 cells	[46]
Coumarin	SC	Aqueous EtOH 80%	50:1	Stirring at 1200 rpm for 3 h	NPs with MD = 165 nm at a high AS/S mixing rate and low c; MPs with MD = 1.2 μm at a low AS/S mixing rate and high c; EE up to 90%; dissolution profile with a burst in the first 30 min + slow release for 5 h	[36]
Curcumin	–	Aqueous EtOH 80%	From 1:1 to 100:1	Stirring for 12 h	NPs with MD = 102 nm; EE up to 94%; dissolution with a burst + SR for 8 h; excellent cellular uptake ability by C6 glioma cells and penetration ability into 3D tumor spheroids	[47]
Curcumin	Tannic acid	Aqueous EtOH 70%	From 5:1 to 100:1	Stirring	NPs with MD = 86–114 nm depending on the c and zein/API ratio; EE up to 98%; controlled release in simulated GI conditions	[48]
Curcumin	–	Aqueous EtOH 75%	10:1	Stirring at 500 rpm for 3 h	NPs with MD = 43 nm; EE = 89%; evaluation of the antioxidant activity	[49]
Curcumin	Chitosan	Aqueous EtOH 75%		Stirring	NPs with MD = 66–170 nm depending on the zein/curcumin ratio; EE up to 95%; evaluation of stability and DPPH assays	[50]
Curcumin	mPEG	Aqueous EtOH 90%	50:1	Stirring at 50 rpm + dialyzing + FD	NPs with MD = 124 nm; EE = 95%; SR up to 24 h; high cell uptake in drug-resistant NCI/ADR-RES cells	[51]
Curcumin	GA	Aqueous EtOH 80%	10:1	Stirring + rotavapor at 35 °C	NPs with an MP lower than 250 nm; EE = 96%; evaluation of the stability of the system under different environmental conditions	[52]
Curcumin	β-CD	Aqueous EtOH 80%	20:1	Rotavapor	Mucoadhesive NPs with MD = 140 nm for the API’s buccal delivery; SR within 10 days	[53]
Curcumin	Pectin	Aqueous EtOH 85%	2.5:1	Stirring + rotavapor	NPs with MD = 230 nm; EE = 90%; 10% of the API released in 2 h (SGF conditions), up to 51% of the API (SIF conditions)—in 10 h	[54]
Curcumin	–	Aqueous EtOH 80%	20:1	Stirring at 1200 rpm + rotavapor	Monomodal NPs with MD = 120 nm; high loading capacity and good chemical stability	[55]
Curcumin	ESP	Aqueous EtOH 85%	From 4:1 to 20:1	Stirring at 800 rpm for 2 h + rotavapor at 45 °C for 35 min	NPs with MD = 253–266 nm depending on the zein/API ratio; EE up to 89%; initial burst release with 50% of the API detected after 2 h SGF digestion; 77% of the API detected after 3 h SIF digestion	[56]
Curcumin	Hya	Aqueous EtOH 70%	20:1	Stirring at 600 rpm for 20 min + rotavapor at 40 °C	NPs with MD = 140 nm; EE = 95%; controlled release in simulated gastroinstestinal digestion	[57]
Curcumin	–	Aqueous EtOH 85%	–	Stirring at 100 rpm for 30 min + rotavapor	NPs with MD = 380–430 nm; EE up to 98%; 40% of the API released in 1.5 h (SGF conditions), more than 95% of the API (SIF conditions)—in 4 h	[58]
Curcumin	EC	Aqueous EtOH 80%	From 16:1 to 80:1	Stirring at 500 rpm for 30 min + rotavapor at 45 °C for 10 min	NPs with MD = 130–179 nm depending on the c, zein/EC, and zein/API ratios; EE up to 82%; pH-dependent release profile; cytotoxicity in HT29 cells	[59]
Curcumin	XG	Aqueous EtOH 80%	–	Stirring at 600 rpm + rotavapor at 40 °C and −0.1 MPa	NPs with MD = 179 nm; EE up to 92%; sustained API release in the GI tract for more than 6 h	[60]
Curcumin + piperine	HA + chitosan	Aqueous EtOH 70%	10:1 + 10:1	Stirring at 600 rpm + rotavapor	NPs with MD = 186 nm; EE = 90% for curcumin and 86% for piperine; piperine was encapsulated in the polysaccharide shell, whereas curcumin was embedded into the hydrophobic cationic zein core	[61]
Docetaxel	CS	Aqueous EtOH 75%	From 3:1 to 5:1	Stirring + dialysis	NPs with MD = 158 nm; EE = 64%; SR in 48–72 h; enhanced tumor accumulation of the NPs was confirmed in PC3 xenograft mice	[62]
DOX	–	Aqueous EtOH 80%	10:1	Stirring at 800 rpm	NPs with MD = 120 nm; EE up to 89%; SR in 15 days	[63]
DOX	SC	Aqueous EtOH 80%	From 20:1 to 60:1	Nitrogen stream + lyophilization in the dark	NPs with MD = 198–244 nm depending on the c and zein/API ratio; EE up to 90%; burst release for 6 h + SR in 100 h; absence of in vitro cytotoxicity in HeLa cells	[64]
DOX hydrochloride	–	Aqueous EtOH 70%	3:1	Stirring and freezing at −20 °C for 12 h	NPs with MD = 150 nm; EE = 21%	[65]
DOX	HA	Aqueous EtOH 70%	3:1	Stirring and freezing at −20 °C for 12 h	NPs with MD = 207 nm; EE = 44%; pH-sensitive SR; in vitro experiments revealed a high cytotoxicity to 4T1; in vivo, decreased DOX cardiotoxicity and liver targeting	[65]
EGCG	Chitosan	Aqueous EtOH 75%	From 14:1 to 72:1	Stirring at 600 rpm for 30 min + rotavapor	NPs with MD = 155–240 nm depending on the c and zein/API ratio; EE up to 81%; release profile with an initial burst effect which occurred within one day, followed by SR over 10 days; significant DPPH scavenging activity	[66]
Felodipine	–	Aqueous EtOH	From 1.5:1 to 4:1	Drying overnight under vacuum at 40 °C	NPs with MD = 150–300 nm depending on the zein/API ratio; EE up to 87%	[67]
Ferulic acid	SC + lysine	Aqueous EtOH 87%		Stirring for 2 h + centrifugation for 20 min	NPs with MD = 199 nm; slow SR for days; in vitro cytotoxicity in Caco-2 and HT29-MTX cells	[68]
Folic acid	–	Aqueous EtOH 70%	1.5:1	Rotavapor for 45 min	NPs with MD = 97 nm; burst release in the first 12 h + SR after 6 days	[69]
Gallic acid	Phospholipid	Aqueous EtOH 90%		Stirring at 600 rpm for 1 h + homogenization at 75,000 rpm	NPs with MD = 269–313 nm; EE up to 65%; fast release within the first 2 h + SR within 24 h; evaluation of the delivery to the activated hepatic stellate cells	[70]
GLI	–	Aqueous EtOH 70%	From 2.5:1 to 50:1	SD (inlet temperature of 90 °C)	NPs with MD = 190 nm; EE = 43%; 5% of the API released in 2 h (SGF conditions), more than 90% of the API (SIF conditions)—in 12 h; in vivo studies using *C. elegans* show a 15% reduction in the fat content	[71]
Glim	–	Aqueous EtOH 90%	From 1:3 to 2:1	Stirring at 2000 rpm for 3 h + rotavapor overnight	MD in the range of 11–603 nm depending on the zein/API ratio, stabilizer type, and concentration; EE up to 63%; API release characterized by an initial burst effect + slow release	[72]
GNA	PDA	Aqueous EtOH 70%	3:1	Stirring at 200 rpm for 2 h + FD at −80 °C for 48 h	NPs with MD = 279–312 nm depending on the presence of PDA; EE up to 82%; SR in more than 72 h; in vitro experiments revealed enhanced cytotoxicity in HepG2; in vivo pharmacokinetic experiments demonstrated tumor-targeting drug delivery	[73]
GNA	–	Aqueous EtOH 70%		Stirring at 30 °C in a ventilated cupboard	NPs with MD = 103 nm; EE = 76%; SR of the API in 36 h; in vivo pharmacokinetic experiments in rats showed an increased bioavailability and prolonged half-life; tissue distribution revealed liver-targeting properties	[74]
Honokiol	PSA	Aqueous EtOH 85%	10:1	Stirring at 1000 rpm for 1 h + rotavapor	NPs with MP = 107 nm; EE = 79%; SR up to 48 h; in vivo experiments in mice revealed enhanced tumor accumulation of the NPs in 4T1, resulting in desirable antitumor efficacy and favorable biosafety	[75]
Hyperoside	Pectin	Aqueous EtOH 85%	From 5:1 to 20:1	Stirring at 800 rpm for 2 h + centrifugation	NPs with MD = 51–298 nm depending on the pectin/zein ratio; EE up to 94%; SR under simulated gastrointestinal conditions	[76]
Indo	Suc	Aqueous EtOH 80%	From 1:2 to 5:1	Stirring for 15 min + centrifugation at 8000 rpm for 15 min	NPs with MD = 112–364 nm depending on the zein/API ratio; EE up to 97%; pH-responsive SR; in vitro evaluation of cytotoxicity in HACAT cells	[77]
Insulin	CSA	Aqueous EtOH 70%	3:1	Stirring at 600 rpm	NPs with MD = 200 nm; EE = 90.5%; dissolution profile with a burst + SR for more than 6 h; in vitro evaluation of cytotoxicity in Caco-2 cells	[78]
Insulin	GT	Aqueous EtOH 55%	10:1	SD	NPs with MD = 222–327 nm depending on the GT/zein ratio; EE = 87%; 30% of the API released in 2 h (SGF conditions), up to 60% of the API (SIF conditions)—in 26 h; in vivo studies using *C. elegans* under high glucose conditions show a 22% reduction of fat storage in the body	[79]
Insulin	GT–PEG	Aqueous EtOH 55%	10:1	SD	NPs with MD = 248 nm; EE = 89%; 28% of the API released in 2 h (SGF conditions), up to 84% of the API (SIF conditions)—in 24 h; in vivo studies using *C. elegans* show a significant reduction in the glucose content and fat accumulated in the body	[80]
Ivermectin	–	Aqueous EtOH 66%	From 2:1 to 20:1	Stirring	MPs with MD = 1 μm; EE up to 69%; SR in 9 days with a burst effect in the first 24 h	[81]
Lovastatin	–	Aqueous EtOH 80%	1:1	Stirring at 2000 rpm for 3 h	NPs with MD = 67 nm; EE = 86%; antiproliferative activity against HepG2 cells	[82]
Lutein	–	Aqueous EtOH 85%	25:1	Stirring at 1000 rpm + rotavapor at 50 °C	NPs with MD = 398 nm; EE = 85%; improved stability in SGF and SIF conditions	[83]
Nic	BSA	Isopropyl alcohol 70%	8:1	Rotavapor for 10–15 min + FD at −53 °C for 48 h	NPs with MD = 173 nm; stable NPs to be used as an injectable nanomedicine with an SR	[84]
Paclitaxel	–	Aqueous EtOH 66%	From 7:1 to 20:1	Stirring at 600 rpm for 12 h	NPs with MD = 265 nm; EE up to 40%; in vitro cytotoxicity in K562 and MCF-7 cells	[85]
Paclitaxel	–	Aqueous EtOH 85%	From 8:1 to 12:1	Stirring at 500 rpm + rotavapor	NPs with MD = 132–495 nm depending on the zein/API ratio; SR without serious bursts in 8 h	[86]
PNS	Lecithin	Aqueous EtOH 80%	From 1:4 to 1:1	Stirring + evaporation at room T for 3 h	NPs with MD = 116–155 nm depending on the zein/API ratio; EE up to 43%; API protected from the degradation of acid and enzymes in the GI tract; good potential to penetrate the mucus layer and enter enterocytes	[87]
PTS	Fucoidan	Aqueous EtOH 75%	10:1	Stirring 1 h + rotavapor	NPs with MD = 74–139 nm depending on the zein/fucoidan ratio; EE up to 95%; 21% of the API released in 2 h (SGF conditions); fast release in 60 min of the SIF conditions; SR up to 46% in 6 h	[88]
Que	HAc	Aqueous EtOH 70%	20:1	Stirring at 600 rpm + rotavapor	NPs with MD = 226 nm; EE = 94%; controlled release of the API	[89]
Que	Chitosan	Aqueous EtOH 70%	20:1	Stirring at 600 rpm + rotavapor at 40 °C	NPs with MD = 330–396 nm depending on the chitosan’s MW; EE up to 95%; delayed release with respect to free API under simulated gastrointestinal conditions	[90]
Que	PGA	Aqueous EtOH 70%	From 2.5:1 to 20:1	Stirring + rotavapor at 45 °C for 35 min	Sub-MPs with MD = 700–900 nm depending on the zein/API ratio; EE up to 96%	[91]
Quercetin	HP-β-CD	Aqueous EtOH 60%	12:1	SD (inlet temperature of 90 °C)	NPs with MD = 294–358 depending on the amount of HP-β-CD; EE = 81%; 15% of the API released in 2 h (SGF conditions), up to 80% of the API (SIF conditions)—in 30 h; after oral administration in rats, high and sustained plasma levels for 30 h	[92]
Rapamycin	Lecithin	Aqueous EtOH 80%		Evaporation at room T for 3 h	NPs with MD = 190 nm; EE = 87%; 65% of the API released in 4 h + 22% SR within the remaining 20 h; enhanced uptake in Caco-2 cells	[93]
Res	PL	Aqueous EtOH 80%	2.5:1	Stirring for 12 h	NPs with MD = 123 nm; EE up to 88%; dissolution profile with a burst for 4 h + SR in 10 h	[94]
Res	–	Aqueous EtOH 65%	6:1	SD (inlet temperature of 90 °C)	NPs with MD = 307 nm; EE = 82%; 20% of the API released in 2 h (SGF conditions), up to 60% of the API—6 h later (SIF conditions); sustained and prolonged release in 48 h; in vivo studies on rats revealed an increased bioavailability and diminished endotoxic symptoms	[95]
Res	–	Aqueous EtOH 70%	From 6:1 to 18:1	Stirring for 3 h + centrifugation for 30–40 min	NPs with MD = 141–187 nm; EE up to 93%; dissolution profile with a burst in 60 min + a plateau up to 24 h; cytotoxicity in Caco-2 and HT29-MTX cells	[96]
Res	–	Aqueous EtOH 70%	6:1	Stirring + SD (inlet temperature of 200 °C)	NPs with MD = 331 nm; EE = 87%; good tolerability and quantifiable plasma levels of the API after administration to 16 volunteers	[97]
Res	Pectin	Aqueous EtOH 88%		Stirring at 500 rpm + rotavapor	Coated NPs with the MD of 250 nm; the release is characterized by a burst effect (38%) in the first 20 min followed by slow release	[98]
Rutin	–	Aqueous EtOH 66%	From 2.5:1 to 10:1	Homogenization at 24,000 rpm for 1 min + stirring at 600 rpm for 12 h	NPs with MD = 103–136 nm depending on the zein/API ratio; EE up to 88%; constant and prolonged release over time; increased protective effect on C-28 and NCTC2544 cells	[99]
Toc	GA	Aqueous EtOH 83%	From 1:1 to 100:1	Stirring at 600 rpm	NPs with MD = 159 nm; EE up to 91%; slow release of Toc in simulated gastrointestinal digestion	[100]
Toc	Chitosan	Aqueous EtOH 75%	From 2.3:1 to 9:1	Stirring at 600 rpm for 1 h	NPs and MP with MD = 211–862 nm depending on the c and zein/API ratio; EE up to 88%; burst release for 1.5 h + SR in 6.5 h; better protection of the TOC release against GI conditions due to chitosan coating	[101]
Vor + Bor	–	Aqueous EtOH 92%		Stirring + dialysis	NPs with MD = 150 nm; EE = 60% for each API; pH-dependent controlled release in 48 h; synergistic toxicity against PC3 and DU145 cells	[102]

**Table 2:** Zein-based particles obtained using the SAS process. AC, acetone; β-car, β-carotene; c, concentration in the liquid solution; δ-toc, δ-tocopherol; DCM, dichloromethane; DIC, diclofenac sodium; DMSO, dimethyl sulfoxide; DR, dissolution rate; EE, encapsulation efficiency; EtOH, ethanol; HCPT, 10-hydroxycamptothecin; MP, microparticles; NP, nanoparticles; P, operating pressure; Rib, riboflavin; T, operating temperature.

Active Principle	Organic Solvent	P (MPa)	T (°C)	c (mg/mL)	Zein/Drug (*w*/*w*)	Results	Reference
Amoxicillin	DMSO	9.0	40–50	50	From 20:1 to 30:1	MPs in the range of 0.26–0.85 μm; EE up to 99.8%; DR about 16 times slower than of the unprocessed API with a burst effect (33%)	[108]
Ampicillin	DMSO	9.0	40–50	50	From 5:1 to 30:1	MPs in the range of 0.31–1.31 μm; EE up to 99.8%; DR about five times slower than of the unprocessed API with a burst effect (10%)	[108]
β-car	Aqueous EtOH 94%	16.0	40	40	1:1	MPs of 11.79 μm	[109]
Cetirizine	DMSO	9.0	40–50	50	From 5:1 to 20:1	MPs in the range of 2.75–8.77 μm; prolonged release (5–6 times longer than the of the unprocessed API) with a burst effect of 10–20%	[110]
δ-toc	Aqueous EtOH 94%	16.0	40	70	From 2:5 to 2:15	MPs in the range of 8.40–14.36 μm	[109]
δ-toc + Rib	Aqueous EtOH 94%	16.0	40	75–175	From 2:5.5 to 2:15.5	MPs in the range of 8.71–16.67 μm	[109]
δ-toc + Rib+ β-car	Aqueous EtOH 94%	16.0	40	200	1:9	MPs of 17.8 μm	[109]
DIC	DMSO	9.0	40	30–50	From 5:1 to 30:1	MPs in the range of 0.31–1.31 μm; delayed release of the API with a burst effect equal to 10% at the optimized operating conditions	[111]
HCPT	DMSO/EtOH *w*/*w* (1/0, ¼, 2/3; 3/2)	8.0–14.0	30–45	6–21	From 5:1 to 20:1	MPs in the range of 0.81–2.99 μm; EE up to 96%; 50% HCPT released in the first 20 h + sustained release up to 92 h; evaluation of in vitro antitumor activity against cancerous cell lines	[112]
Ketotifen	DMSO	9.0	40–50	50	From 5:1 to 20:1	MPs in the range of 0.72–2.23 μm; prolonged release with a burst effect equal to 10% at the optimized temperature	[110]
Lutein	AC/DMSO 7/3 *v*/*v*	10.0–15.0	32–45	10–20	From 12:1 to 24:1	NPs in the range of 198–355 nm; EE up to 83%; controlled release without a burst effect	[113]
Lysozyme	Aqueous EtOH 90%	10.0	40	5	50:1	MPs in the range of 1–50 μm; EE = 47%; sustained release	[114]
Melatonin	DCM/EtOH 7/5 *v*/*v*	8.0–16.0	35–55	10.5–11	From 10:1 to 20:1	NPs in the range of 69–354 nm; EE up to 80%; controlled release without a burst effect	[115]
Rib	Aqueous EtOH 94%	16.0	40	22.5–27.5	From 2.6:1 to 8:1	MPs in the range of 11.24–14.21 μm	[109]

## Data Availability

Not applicable.

## References

[1] Landry J., Moureaux T. (1980). Distribution and amino acid composition of protein groups located in different histological parts of maize grain. J. Agric. Food Chem..

[2] Malekzad H., Mirshekari H., Sahandi Zangabad P., Moosavi Basri S., Baniasadi F., Sharifi Aghdam M., Karimi M., Hamblin M.R. (2018). Plant protein-based hydrophobic fine and ultrafine carrier particles in drug delivery systems. Crit. Rev. Biotechnol..

[3] Coleman C.E., Larkins B.A. (1999). The prolamins of maize. Seed Proteins.

[4] Lawton J.W. (2002). Zein: A history of processing and use. Cereal Chem..

[5] Esen A. (1987). A proposed nomenclature for the alcohol-soluble proteins (zeins) of maize (*Zea mays* L.). J. Cereal Sci..

[6] Mahanty A., Abbasi Y.F., Bera H., Chakraborty M., Al Maruf M.A. (2021). Zein-based nanomaterials in drug delivery and biomedical applications. Biopolymer-Based Nanomaterials in Drug Delivery and Biomedical Applications.

[7] Kasaai M.R. (2018). Zein and zein-based nano-materials for food and nutrition applications: A review. Trends Food Sci. Technol..

[8] Bai J., Alleyne V., Hagenmaier R.D., Mattheis J.P., Baldwin E.A. (2003). Formulation of zein coatings for apples (Malus domestica Borkh). Postharvest Biol. Technol..

[9] Drago E., Franco P., Campardelli R., De Marco I., Perego P. (2022). Zein electrospun fibers purification and vanillin impregnation in a one-step supercritical process to produce safe active packaging. Food Hydrocoll..

[10] Wei Y., Yao J., Shao Z., Chen X. (2020). Water-Resistant Zein-Based Adhesives. ACS Sustain. Chem. Eng..

[11] Parris N., Dickey L.C. (2003). Adhesive properties of corn zein formulations on glass surfaces. J. Agric. Food Chem..

[12] Zhang M., Reitmeier C.A., Hammond E.G., Myers D.J. (1997). Production of textile fibers from zein and a soy protein-zein blend. Cereal Chem..

[13] Paliwal R., Palakurthi S. (2014). Zein in controlled drug delivery and tissue engineering. J. Control. Release.

[14] Zhang Y., Cui L., Chen Y., Zhang H., Zhong J., Sun Y., Shi N., Li C., Kong W. (2015). Zein-based nanofibres for drug delivery: Classes and current applications. Curr. Pharm. Des..

[15] Luo Y., Wang Q. (2014). Zein-based micro- and nano-particles for drug and nutrient delivery: A review. J. Appl. Polym. Sci..

[16] Del Nobile M.A., Conte A., Incoronato A.L., Panza O. (2008). Antimicrobial efficacy and release kinetics of thymol from zein films. J. Food Eng..

[17] Miyoshi T., Toyohara K., Minematsu H. (2005). Preparation of ultrafine fibrous zein membranes via electrospinning. Polym. Int..

[18] Tu J., Wang H., Li H., Dai K., Wang J., Zhang X. (2009). The in vivo bone formation by mesenchymal stem cells in zein scaffolds. Biomaterials.

[19] Tortorella S., Maturi M., Vetri Buratti V., Vozzolo G., Locatelli E., Sambri L., Comes Franchini M. (2021). Zein as a versatile biopolymer: Different shapes for different biomedical applications. RSC Adv..

[20] Demir M., Ramos-Rivera L., Silva R., Nazhat S.N., Boccaccini A.R. (2017). Zein-based composites in biomedical applications. J. Biomed. Mater. Res. Part A.

[21] Dong J., Sun Q., Wang J.Y. (2004). Basic study of corn protein, zein, as a biomaterial in tissue engineering, surface morphology and biocompatibility. Biomaterials.

[22] Campardelli R., Pettinato M., Drago E., Perego P. (2021). Production of Vanillin-Loaded Zein Sub-micron Electrospun Fibers for Food Packaging Applications. Chem. Eng. Technol..

[23] Berardi A., Bisharat L., AlKhatib H.S., Cespi M. (2018). Zein as a Pharmaceutical Excipient in Oral Solid Dosage Forms: State of the Art and Future Perspectives. AAPS PharmSciTech.

[24] Raza A., Hayat U., Bilal M., Iqbal H.M.N., Wang J.Y. (2020). Zein-based micro- and nano-constructs and biologically therapeutic cues with multi-functionalities for oral drug delivery systems. J. Drug Deliv. Sci. Technol..

[25] Tran P.H.L., Duan W., Lee B.J., Tran T.T.D. (2019). The use of zein in the controlled release of poorly water-soluble drugs. Int. J. Pharm..

[26] Sivalingan G., GNK G., Chandrasekaran M. (2020). Multiparticulate Drug Delivery System. Res. J. Pharm. Technol..

[27] Padrela L., Rodrigues M.A., Duarte A., Dias A.M.A., Braga M.E.M., de Sousa H.C. (2018). Supercritical carbon dioxide-based technologies for the production of drug nanoparticles/nanocrystals—A comprehensive review. Adv. Drug Deliv. Rev..

[28] Wais U., Jackson A.W., He T., Zhang H. (2016). Nanoformulation and encapsulation approaches for poorly water-soluble drug nanoparticles. Nanoscale.

[29] Möschwitzer J.P. (2013). Drug nanocrystals in the commercial pharmaceutical development process. Int. J. Pharm..

[30] Khadka P., Ro J., Kim H., Kim I., Kim J.T., Kim H., Cho J.M., Yun G., Lee J. (2014). Pharmaceutical particle technologies: An approach to improve drug solubility, dissolution and bioavailability. Asian J. Pharm. Sci..

[31] Rasenack N., Müller B.W. (2004). Micron-Size Drug Particles: Common and Novel Micronization Techniques. Pharm. Dev. Technol..

[32] Verma S., Gokhale R., Burgess D.J. (2009). A comparative study of top-down and bottom-up approaches for the preparation of micro/nanosuspensions. Int. J. Pharm..

[33] Huang Q., Yu H., Ru Q. (2010). Bioavailability and Delivery of Nutraceuticals Using Nanotechnology. J. Food Sci..

[34] Zhong Q., Jin M. (2009). Zein nanoparticles produced by liquid-liquid dispersion. Food Hydrocoll..

[35] Podaralla S., Perumal O. (2012). Influence of formulation factors on the preparation of zein nanoparticles. AAPS PharmSciTech.

[36] Li F., Chen Y., Liu S., Qi J., Wang W., Wang C., Zhong R., Chen Z., Li X., Guan Y. (2017). Size-controlled fabrication of zein nano/microparticles by modified anti-solvent precipitation with/without sodium caseinate. Int. J. Nanomed..

[37] Reboredo C., González-Navarro C.J., Martínez-Oharriz C., Martínez-López A.L., Irache J.M. (2021). Preparation and evaluation of PEG-coated zein nanoparticles for oral drug delivery purposes. Int. J. Pharm..

[38] Li S., Zhao Y. (2017). Preparation of zein nanoparticles by using solution-enhanced dispersion with supercritical co2and elucidation with computational fluid dynamics. Int. J. Nanomed..

[39] Patel A.R., Bouwens E.C.M., Velikov K.P. (2010). Sodium caseinate stabilized zein colloidal particles. J. Agric. Food Chem..

[40] Lai L.F., Guo H.X. (2011). Preparation of new 5-fluorouracil-loaded zein nanoparticles for liver targeting. Int. J. Pharm..

[41] Boateng-Marfo Y., Dong Y., Ng W.K., Lin H.S. (2021). Artemether-loaded zein nanoparticles: An innovative intravenous dosage form for the management of severe malaria. Int. J. Mol. Sci..

[42] Hashem F.M., Al-Sawahli M.M., Nasr M., Ahmed O.A.A. (2015). Optimized zein nanospheres for improved oral bioavailability of atorvastatin. Int. J. Nanomed..

[43] Gagliardi A., Paolino D., Iannone M., Palma E., Fresta M., Cosco D. (2018). Sodium deoxycholate-decorated zein nanoparticles for a stable colloidal drug delivery system. Int. J. Nanomed..

[44] Gagliardi A., Voci S., Giuliano E., Salvatici M.C., Celano M., Fresta M., Cosco D. (2021). Phospholipid/zein hybrid nanoparticles as promising carriers for the protection and delivery of all-trans retinoic acid. Mater. Sci. Eng. C.

[45] Wusigale, Wang T., Hu Q., Xue J., Khan M.A., Liang L., Luo Y. (2021). Partition and stability of folic acid and caffeic acid in hollow zein particles coated with chitosan. Int. J. Biol. Macromol..

[46] Shinde P., Agraval H., Srivastav A.K., Yadav U.C.S., Kumar U. (2020). Physico-chemical characterization of carvacrol loaded zein nanoparticles for enhanced anticancer activity and investigation of molecular interactions between them by molecular docking. Int. J. Pharm..

[47] Zhang H., Van Os W.L., Tian X., Zu G., Ribovski L., Bron R., Bussmann J., Kros A., Liu Y., Zuhorn I.S. (2021). Development of curcumin-loaded zein nanoparticles for transport across the blood-brain barrier and inhibition of glioblastoma cell growth. Biomater. Sci..

[48] Hu S., Wang T., Fernandez M.L., Luo Y. (2016). Development of tannic acid cross-linked hollow zein nanoparticles as potential oral delivery vehicles for curcumin. Food Hydrocoll..

[49] Liu J., Li Y., Zhang H., Liu S., Yang M., Cui M., Zhang T., Yu Y., Xiao H., Du Z. (2022). Fabrication, characterization and functional attributes of zein-egg white derived peptides (EWDP)-chitosan ternary nanoparticles for encapsulation of curcumin: Role of EWDP. Food Chem..

[50] Liang H., Zhou B., He L., An Y., Lin L., Li Y., Liu S., Chen Y., Li B. (2015). Fabrication of zein/quaternized chitosan nanoparticles for the encapsulation and protection of curcumin. RSC Adv..

[51] Podaralla S., Averineni R., Alqahtani M., Perumal O. (2012). Synthesis of novel biodegradable methoxy poly(ethylene glycol)-zein micelles for effective delivery of curcumin. Mol. Pharm..

[52] Chen G., Fu Y., Niu F., Zhang H., Li X., Li X. (2019). Evaluation of the colloidal/chemical performance of core-shell nanoparticle formed by zein and gum Arabic. Colloids Surf. A Physicochem. Eng. Asp..

[53] Esposito D., Conte C., d’Angelo I., Miro A., Ungaro F., Quaglia F. (2020). Mucoadhesive zein/beta-cyclodextrin nanoparticles for the buccal delivery of curcumin. Int. J. Pharm..

[54] Cai T., Xiao P., Yu N., Zhou Y., Mao J., Peng H., Deng S. (2020). A novel pectin from Akebia trifoliata var. australis fruit peel and its use as a wall-material to coat curcumin-loaded zein nanoparticle. Int. J. Biol. Macromol..

[55] Zou L., Zheng B., Zhang R., Zhang Z., Liu W., Liu C., Xiao H., McClements D.J. (2016). Enhancing the bioaccessibility of hydrophobic bioactive agents using mixed colloidal dispersions: Curcumin-loaded zein nanoparticles plus digestible lipid nanoparticles. Food Res. Int..

[56] Zhang T., Yu S., Tang X., Ai C., Chen H., Lin J., Meng H., Guo X. (2022). Ethanol-soluble polysaccharide from sugar beet pulp for stabilizing zein nanoparticles and improving encapsulation of curcumin. Food Hydrocoll..

[57] Chen S., Han Y., Sun C., Dai L., Yang S., Wei Y., Mao L., Yuan F., Gao Y. (2018). Effect of molecular weight of hyaluronan on zein-based nanoparticles: Fabrication, structural characterization and delivery of curcumin. Carbohydr. Polym..

[58] Sun X., Pan C., Ying Z., Yu D., Duan X., Huang F., Ling J., Ouyang X.K. (2020). Stabilization of zein nanoparticles with k-carrageenan and tween 80 for encapsulation of curcumin. Int. J. Biol. Macromol..

[59] Hasankhan S., Tabibiazar M., Hosseini S.M., Ehsani A., Ghorbani M. (2020). Fabrication of curcumin-zein-ethyl cellulose composite nanoparticles using antisolvent co-precipitation method. Int. J. Biol. Macromol..

[60] Zhang D., Jiang F., Ling J., Ouyang X.K., Wang Y.G. (2021). Delivery of curcumin using a zein-xanthan gum nanocomplex: Fabrication, characterization, and in vitro release properties. Colloids Surf. B.

[61] Chen S., McClements D.J., Jian L., Han Y., Dai L., Mao L., Gao Y. (2019). Core–Shell Biopolymer Nanoparticles for Co-Delivery of Curcumin and Piperine: Sequential Electrostatic Deposition of Hyaluronic Acid and Chitosan Shells on the Zein Core. ACS Appl. Mater. Interfaces.

[62] Lee H.S., Kang N.W., Kim H., Kim D.H., Chae J.W., Lee W., Song G.Y., Cho C.W., Kim D.D., Lee J.Y. (2021). Chondroitin sulfate-hybridized zein nanoparticles for tumor-targeted delivery of docetaxel. Carbohydr. Polym..

[63] Arunkumar P., Indulekha S., Vijayalakshmi S., Srivastava R. (2017). In vitro comparative studies of Zein nanoparticles and composite Chitosan thermogels based injectable formulation of Doxorubicin. J. Drug Deliv. Sci. Technol..

[64] Dong F., Dong X., Zhou L., Xiao H., Ho P.Y., Wong M.S., Wang Y. (2016). Doxorubicin-loaded biodegradable self-assembly zein nanoparticle and its anti-cancer effect: Preparation, in vitro evaluation, and cellular uptake. Colloids Surf. B.

[65] Zha L., Wang B., Qian J., Fletcher B., Zhang C., Dong Q., Chen W., Hong L. (2020). Preparation, characterization and preliminary pharmacokinetic study of pH-sensitive Hydroxyapatite/Zein nano-drug delivery system for doxorubicin hydrochloride. J. Pharm. Pharmacol..

[66] Liang J., Yan H., Wang X., Zhou Y., Gao X., Puligundla P., Wan X. (2017). Encapsulation of epigallocatechin gallate in zein/chitosan nanoparticles for controlled applications in food systems. Food Chem..

[67] Ren F., Fu J., Xiong H., Cui L., Ren G., Guan H., Jing Q. (2018). Complexes of Felodipine Nanoparticles With Zein Prepared Using a Dual Shift Technique. J. Pharm. Sci..

[68] Heep G., Almeida A., Marcano R., Vieira D., Mainardes R.M., Khalil N.M., Sarmento B. (2019). Zein-casein-lysine multicomposite nanoparticles are effective in modulate the intestinal permeability of ferulic acid. Int. J. Biol. Macromol..

[69] Chuacharoen T., Sabliov C.M. (2017). Zein nanoparticles as delivery systems for covalently linked and physically entrapped folic acid. J. Nanoparticle Res..

[70] Radwan S.A.A., El-Maadawy W.H., Yousry C., Elmeshad A.N., Shoukri R.A. (2020). Zein/phospholipid composite nanoparticles for successful delivery of gallic acid into ahscs: Influence of size, surface charge, and vitamin a coupling. Int. J. Nanomed..

[71] Lucio D., Martínez-Ohárriz M.C., Jaras G., Aranaz P., González-Navarro C.J., Radulescu A., Irache J.M. (2017). Optimization and evaluation of zein nanoparticles to improve the oral delivery of glibenclamide. In vivo study using *C. elegans*. Eur. J. Pharm. Biopharm..

[72] Ahmed O.A.A., Zidan A.S., Khayat M. (2016). Mechanistic analysis of zein nanoparticles/PLGA triblock in situ forming implants for glimepiride. Int. J. Nanomed..

[73] Zha L., Qian J., Wang B., Liu H., Zhang C., Dong Q., Chen W., Hong L. (2020). In vitro/in vivo evaluation of pH-sensitive Gambogenic acid loaded Zein nanoparticles with polydopamine coating. Int. J. Pharm..

[74] Cheng W., Wang B., Zhang C., Dong Q., Qian J., Zha L., Chen W., Hong L. (2019). Preparation and preliminary pharmacokinetics study of GNA-loaded zein nanoparticles. J. Pharm. Pharmacol..

[75] Zhang Q., Li D., Guan S., Liu D., Wang J., Xing G., Yue L., Cai D. (2022). Tumor-targeted delivery of honokiol via polysialic acid modified zein nanoparticles prevents breast cancer progression and metastasis. Int. J. Biol. Macromol..

[76] Wang X., Peng F., Liu F., Xiao Y., Li F., Lei H., Wang J., Li M., Xu H. (2020). Zein-pectin composite nanoparticles as an efficient hyperoside delivery system: Fabrication, characterization, and in vitro release property. LWT.

[77] Zhu W., Huang W., Ye L., Deng Y., Xie Q., Jiang Y. (2020). Facile preparation of succinylated-zein-ZIF-8 hybrid for enhanced stability and pH-responsive drug delivery. Chem. Eng. Sci..

[78] Ji N., Hong Y., Gu Z., Cheng L., Li Z., Li C. (2018). Preparation and Characterization of Insulin-Loaded Zein/Carboxymethylated Short-Chain Amylose Complex Nanoparticles. J. Agric. Food Chem..

[79] Inchaurraga L., Martínez-López A.L., Martin-Arbella N., Irache J.M. (2020). Zein-based nanoparticles for the oral delivery of insulin. Drug Deliv. Transl. Res..

[80] Martínez-López A.L., González-Navarro C.J., Vizmanos J.L., Irache J.M. (2021). Zein-based nanocarriers for the oral delivery of insulin. In vivo evaluation in *Caenorhabditis elegans*. Drug Deliv. Transl. Res..

[81] Liu X., Sun Q., Wang H., Zhang L., Wang J.Y. (2005). Microspheres of corn protein, zein, for an ivermectin drug delivery system. Biomaterials.

[82] Alhakamy N.A., Ahmed O.A.A., Aldawsari H.M., Alfaifi M.Y., Eid B.G., Abdel-Naim A.B., Fahmy U.A. (2019). Encapsulation of lovastatin in zein nanoparticles exhibits enhanced apoptotic activity in hepg2 cells. Int. J. Mol. Sci..

[83] Jiao Y., Zheng X., Chang Y., Li D., Sun X., Liu X. (2018). Zein-derived peptides as nanocarriers to increase the water solubility and stability of lutein. Food Funct..

[84] Sanoj Rejinold N., Choi G., Piao H., Choy J.H. (2021). Bovine serum albumin-coated niclosamide-zein nanoparticles as potential injectable medicine against covid-19. Materials.

[85] Gagliardi A., Bonacci S., Paolino D., Celia C., Procopio A., Fresta M., Cosco D. (2019). Paclitaxel-loaded sodium deoxycholate-stabilized zein nanoparticles: Characterization and in vitro cytotoxicity. Heliyon.

[86] Li S., Wang X., Li W., Yuan G., Pan Y., Chen H. (2016). Preparation and characterization of a novel conformed bipolymer paclitaxel-nanoparticle using tea polysaccharides and zein. Carbohydr. Polym..

[87] Fu W., Liang Y., Xie Z., Wu H., Zhang Z., Lv H. (2021). Preparation and evaluation of lecithin/zein hybrid nanoparticles for the oral delivery of Panax notoginseng saponins. Eur. J. Pharm. Sci..

[88] Liu Q., Chen J., Qin Y., Jiang B., Zhang T. (2020). Zein/fucoidan-based composite nanoparticles for the encapsulation of pterostilbene: Preparation, characterization, physicochemical stability, and formation mechanism. Int. J. Biol. Macromol..

[89] Chen S., Han Y., Wang Y., Yang X., Sun C., Mao L., Gao Y. (2019). Zein-hyaluronic acid binary complex as a delivery vehicle of quercetagetin: Fabrication, structural characterization, physicochemical stability and in vitro release property. Food Chem..

[90] Chen S., Ma X., Han Y., Wei Y., Guo Q., Yang S., Zhang Y., Liao W., Gao Y. (2020). Effect of chitosan molecular weight on zein-chitosan nanocomplexes: Formation, characterization, and the delivery of quercetagetin. Int. J. Biol. Macromol..

[91] Sun C., Dai L., Gao Y. (2016). Binary Complex Based on Zein and Propylene Glycol Alginate for Delivery of Quercetagetin. Biomacromolecules.

[92] Penalva R., González-Navarro C.J., Gamazo C., Esparza I., Irache J.M. (2017). Zein nanoparticles for oral delivery of quercetin: Pharmacokinetic studies and preventive anti-inflammatory effects in a mouse model of endotoxemia. Nanomed. Nanotechnol. Biol. Med..

[93] Xie Z., Zhang Z., Lv H. (2019). Rapamycin loaded TPGS-Lecithins-Zein nanoparticles based on core-shell structure for oral drug administration. Int. J. Pharm..

[94] Huang W., Li S., Li Z., Zhu W., Lu S., Jiang Y. (2019). Development of a resveratrol–zein–dopamine–lecithin delivery system with enhanced stability and mucus permeation. J. Mater. Sci..

[95] Penalva R., Esparza I., Larraneta E., González-Navarro C.J., Gamazo C., Irache J.M. (2015). Zein-Based Nanoparticles Improve the Oral Bioavailability of Resveratrol and Its Anti-inflammatory Effects in a Mouse Model of Endotoxic Shock. J. Agric. Food Chem..

[96] Nunes R., Baião A., Monteiro D., das Neves J., Sarmento B. (2020). Zein nanoparticles as low-cost, safe, and effective carriers to improve the oral bioavailability of resveratrol. Drug Deliv. Transl. Res..

[97] Brotons-Canto A., Gonzalez-Navarro C.J., Gurrea J., González-Ferrero C., Irache J.M. (2020). Zein nanoparticles improve the oral bioavailability of resveratrol in humans. J. Drug Deliv. Sci. Technol..

[98] Contado C., Caselotto L., Mello P., Maietti A., Marvelli L., Marchetti N., Dalpiaz A. (2020). Design and formulation of Eudragit-coated zein/pectin nanoparticles for the colon delivery of resveratrol. Eur. Food Res. Technol..

[99] Gagliardi A., Paolino D., Costa N., Fresta M., Cosco D. (2021). Zein- vs PLGA-based nanoparticles containing rutin: A comparative investigation. Mater. Sci. Eng. C.

[100] Li J., Xu X., Chen Z., Wang T., Wang L., Zhong Q. (2018). Biological macromolecule delivery system fabricated using zein and gum arabic to control the release rate of encapsulated tocopherol during in vitro digestion. Food Res. Int..

[101] Luo Y., Zhang B., Whent M., Yu L., Wang Q. (2011). Preparation and characterization of zein/chitosan complex for encapsulation of α-tocopherol, and its in vitro controlled release study. Colloids Surf. B.

[102] Thapa R.K., Nguyen H.T., Jeong J.H., Shin B.S., Ku S.K., Choi H.G., Yong C.S., Kim J.O. (2017). Synergistic anticancer activity of combined histone deacetylase and proteasomal inhibitor-loaded zein nanoparticles in metastatic prostate cancers. Nanomed. Nanotechnol. Biol. Med..

[103] Franco P., De Marco I. (2020). Eudragit: A novel carrier for controlled drug delivery in supercritical antisolvent coprecipitation. Polymers.

[104] Franco P., De Marco I. (2020). Supercritical Antisolvent Process for Pharmaceutical Applications: A Review. Processes.

[105] Montes A., Gordillo M.D., Pereyra C., Martínez de la Ossa E.J. (2011). Co-precipitation of amoxicillin and ethyl cellulose microparticles by supercritical antisolvent process. J. Supercrit. Fluids.

[106] Reverchon E., Adami R., De Marco I., Laudani C., Spada A. (2005). Pigment Red 60 micronization using supercritical fluids based techniques. J. Supercrit. Fluids.

[107] Franco P., De Marco I. (2020). Supercritical antisolvent coprecipitation in the pharmaceutical field: Different polymeric carriers for different drug releases. Can. J. Chem. Eng..

[108] Franco P., Reverchon E., De Marco I. (2019). Production of zein/antibiotic microparticles by supercritical antisolvent coprecipitation. J. Supercrit. Fluids.

[109] Rosa M.T.M.G., Alvarez V.H., Albarelli J.Q., Santos D.T., Meireles M.A.A., Saldaña M.D.A. (2019). Supercritical anti-solvent process as an alternative technology for vitamin complex encapsulation using zein as wall material: Technical-economic evaluation. J. Supercrit. Fluids.

[110] Franco P., De Marco I. (2021). Controlled-release antihistamines using supercritical antisolvent process. J. Supercrit. Fluids.

[111] Franco P., Reverchon E., De Marco I. (2018). Zein/diclofenac sodium coprecipitation at micrometric and nanometric range by supercritical antisolvent processing. J. CO2 Util..

[112] Liu G., Li S., Huang Y., Wang H., Jiang Y. (2016). Incorporation of 10-hydroxycamptothecin nanocrystals into zein microspheres. Chem. Eng. Sci..

[113] Hu D., Lin C., Liu L., Li S., Zhao Y. (2012). Preparation, characterization, and in vitro release investigation of lutein/zein nanoparticles via solution enhanced dispersion by supercritical fluids. J. Food Eng..

[114] Zhong Q., Jin M., Davidson P.M., Zivanovic S. (2009). Sustained release of lysozyme from zein microcapsules produced by a supercritical anti-solvent process. Food Chem..

[115] Li S., Zhao Y. (2017). Preparation of Melatonin-Loaded Zein Nanoparticles using Supercritical CO2 Antisolvent and in vitro Release Evaluation. Int. J. Food Eng..

[116] Liu G., Pang J., Huang Y., Xie Q., Guan G., Jiang Y. (2017). Self-Assembled Nanospheres of Folate-Decorated Zein for the Targeted Delivery of 10-Hydroxycamptothecin. Ind. Eng. Chem. Res..

[117] Palazzo I., Campardelli R., Scognamiglio M., Reverchon E. (2019). Zein/luteolin microparticles formation using a supercritical fluids assisted technique. Powder Technol..

[118] Wang H.J., Lin Z.X., Liu X.M., Sheng S.Y., Wang J.Y. (2005). Heparin-loaded zein microsphere film and hemocompatibility. J. Control. Release.

[119] Muthuselvi L., Dhathathreyan A. (2006). Simple coacervates of zein to encapsulate Gitoxin. Colloids Surf. B.

[120] Suzuki T., Sato E., Matsuda Y., Tada H., Unno K., Kato T. (1989). Preparation of Zein Microspheres Conjugated with Antitumor Drugs Available for Selective Cancer Chemotherapy and Development of a Simple Colorimetric Determination of Drugs in Microspheres. Chem. Pharm. Bull..

[121] Hurtado-López P., Murdan S. (2005). Formulation and characterisation of zein microspheres as delivery vehicles. J. Drug Deliv. Sci. Technol..

[122] Hurtado-López P., Murdan S. (2006). An investigation into the adjuvanticity and immunogenicity of zein microspheres being researched as drug and vaccine carriers. J. Pharm. Pharmacol..

[123] Regier M.C., Taylor J.D., Borcyk T., Yang Y., Pannier A.K. (2012). Fabrication and characterization of DNA-loaded zein nanospheres. J. Nanobiotechnology.

[124] Karthikeyan K., Lakra R., Rajaram R., Korrapati P.S. (2012). Development and characterization of zein-based micro carrier system for sustained delivery of aceclofenac sodium. AAPS PharmSciTech.

[125] Karthikeyan K., Vijayalakshmi E., Korrapati P.S. (2014). Selective interactions of zein microspheres with different class of drugs: An in vitro and in silico analysis. AAPS PharmSciTech.

[126] Coelho S.C., Laget S., Benaut P., Rocha F., Estevinho B.N. (2021). A new approach to the production of zein microstructures with vitamin B12, by electrospinning and spray drying techniques. Powder Technol..

[127] Mahalakshmi L., Leena M.M., Moses J.A., Anandharamakrishnan C. (2020). Micro- and nano-encapsulation of β-carotene in zein protein: Size-dependent release and absorption behavior. Food Funct..

[128] Sousa F.F.O., Luzardo-Álvarez A., Pérez-Estévéz A., Seoane-Prado R., Blanco-Méndez J. (2010). Development of a novel AMX-loaded PLGA/zein microsphere for root canal disinfection. Biomed. Mater..

[129] De Sousa F.O., Blanco-Méndez J., Pérez-Estévez A., Seoane-Prado R., Luzardo-Álvarez A. (2012). Effect of zein on biodegradable inserts for the delivery of tetracycline within periodontal pockets. J. Biomater. Appl..

[130] Tavares W.D.S., Pena G.R., Martin-Pastor M., Sousa F.F.O.D. (2021). Design and characterization of ellagic acid-loaded zein nanoparticles and their effect on the antioxidant and antibacterial activities. J. Mol. Liq..

[131] Weissmueller N.T., Lu H.D., Hurley A., Prud’Homme R.K. (2016). Nanocarriers from GRAS Zein Proteins to Encapsulate Hydrophobic Actives. Biomacromolecules.

[132] Rodrigues D.A., Miguel S.P., Loureiro J., Ribeiro M., Roque F., Coutinho P. (2021). Oromucosal alginate films with zein nanoparticles as a novel delivery system for digoxin. Pharmaceutics.

[133] Yuan Y., Xiao J., Zhang P., Ma M., Wang D., Xu Y. (2021). Development of pH-driven zein/tea saponin composite nanoparticles for encapsulation and oral delivery of curcumin. Food Chem..

[134] Sabra S.A., Elzoghby A.O., Sheweita S.A., Haroun M., Helmy M.W., Eldemellawy M.A., Xia Y., Goodale D., Allan A.L., Rohani S. (2018). Self-assembled amphiphilic zein-lactoferrin micelles for tumor targeted co-delivery of rapamycin and wogonin to breast cancer. Eur. J. Pharm. Biopharm..

[135] Vozza G., Khalid M., Byrne H.J., Ryan S.M., Frias J.M. (2019). Nutraceutical formulation, characterisation, and in-vitro evaluation of methylselenocysteine and selenocystine using food derived chitosan:zein nanoparticles. Food Res. Int..

[136] Vozza G., Danish M., Byrne H.J., Frías J.M., Ryan S.M. (2018). Application of Box-Behnken experimental design for the formulation and optimisation of selenomethionine-loaded chitosan nanoparticles coated with zein for oral delivery. Int. J. Pharm..

[137] Farris E., Brown D.M., Ramer-Tait A.E., Pannier A.K. (2017). Chitosan-zein nano-in-microparticles capable of mediating in vivo transgene expression following oral delivery. J. Control. Release.

